# Preoperative gut microbial and metabolic signatures associated with postoperative Hirschsprung-associated enterocolitis in short-segment Hirschsprung disease

**DOI:** 10.3389/fmicb.2026.1911505

**Published:** 2026-09-30

**Authors:** Minzhong Zhang, Yiming Gong, Tian Zhang

**Affiliations:** 1 Department of Pediatric General Surgery, Xinhua Hospital, Shanghai Jiao Tong University School of Medicine, Shanghai, China; 2 Shanghai Key Laboratory of Pediatric Gastroenterology and Nutrition, Shanghai, China

**Keywords:** gut microbiome, Hirschsprung disease, Hirschsprung-associated enterocolitis, metabolomics, short-chain fatty acids

## Abstract

**Introduction:**

Hirschsprung-associated enterocolitis (HAEC) is a major postoperative complication of Hirschsprung disease (HSCR), but whether preoperative gut microbial and metabolic alterations are associated with its subsequent development remains unclear. This prospective cohort study investigated whether preoperative gut microbial and metabolic profiles are associated with postoperative HAEC in children with short-segment Hirschsprung disease (S-HSCR).

**Methods:**

Sixty-two children with S-HSCR were enrolled, and preoperative fecal samples were collected before pull-through surgery for 16S rRNA gene sequencing, targeted short-chain fatty acid (SCFA) analysis, and untargeted metabolomic profiling. Patients were followed for 3 years after surgery and classified according to the occurrence of postoperative HAEC.

**Results:**

During follow-up, 27 patients developed postoperative HAEC. Compared with patients who remained HAEC-free, those who subsequently developed HAEC showed reduced microbial diversity, a lower Gut Microbiome Health Index, a higher Microbiome Dysbiosis Index, and distinct microbial community composition before surgery. Differential abundance analyses identified depletion of several anaerobic commensals and potential SCFA-producing taxa, together with enrichment of potentially pro-inflammatory taxa such as *Escherichia-Shigella*. Co-occurrence network analysis revealed reduced microbial interaction complexity and connectivity in the HAEC cohort. An exploratory random forest model based on preoperative microbial signatures achieved an area under the curve of 0.80 for predicting postoperative HAEC. Children who later developed HAEC also had significantly lower fecal levels of butyric acid, valeric acid, and total SCFAs. Exploratory untargeted metabolomic analysis yielded preliminary signals across several metabolic pathways, including lysine-related metabolism.

**Discussion:**

Children with S-HSCR who subsequently develop postoperative HAEC already exhibit distinct preoperative microbial dysbiosis and metabolic alterations. These findings suggest that early-life disruption of the gut microbiota and host-microbe metabolic balance may contribute to HAEC susceptibility.

## 1 Introduction

Hirschsprung disease (HSCR) is a congenital disorder characterized by abnormal development of the enteric nervous system (ENS) and requires surgical resection of the aganglionic segment. While the majority of children recover following definitive surgery, approximately one-third still experience postoperative complications. Among these, Hirschsprung-associated enterocolitis (HAEC) is particularly common and can be life-threatening. Strategies to predict and prevent postoperative HAEC are still limited (On Behalf Of The American Pediatric Surgical Association et al., 2017; Montalva et al., 2023).

Growing evidence indicates that HAEC is not just a result of mechanical stasis but a multifactorial disorder involving ENS dysfunction, compromised epithelial barrier integrity, mucosal immune dysregulation, and an imbalance in the gut microbiome. Alterations in the gut microbiota have been identified as one of the most reproducible biological features associated with HAEC pathogenesis (Zhao et al., 2026). However, much of the clinical evidence is derived from fecal or mucosal samples collected during or after HAEC episodes or after pull-through surgery, which makes it difficult to determine whether microbial dysbiosis is already present before surgery and precedes HAEC, or whether it instead reflects intestinal inflammation, surgery, or treatment-related exposures (Chantakhow et al., 2021; Arnaud et al., 2022; Biassoni et al., 2022).

Accumulating evidence indicates that early-life gut microbial exposure modulates ENS development and may exert durable effects on disease susceptibility (Zhang et al., 2025). In HSCR, ENS abnormalities are present from birth and may influence luminal stasis, mucosal signaling, and microbial maturation from the earliest postnatal stages (Liu et al., 2024). HAEC-associated microbial dysbiosis appears to be most pronounced in younger children, and postoperative patients with HSCR have been shown to exhibit persistent microbial deviations and subclinical inflammation, even in the absence of overt HAEC (Neuvonen et al., 2018; Arnaud et al., 2022; Murphy et al., 2025). Moreover, a prospective study demonstrated that microbial dysbiosis may precede the clinical onset of HAEC, with the mucosal microbiome profile at the time of pull-through surgery closely associated with subsequent HAEC development (Tang et al., 2020). These findings suggest that early, pre-existing deviations in the gut microenvironment may serve as an important predisposing factor for postoperative HAEC.

Beyond taxonomic alterations, emerging studies suggest that metabolic abnormalities may promote colonic ecological dysregulation and contribute to HAEC development (Demehri et al., 2016; Plekhova et al., 2021). However, available metabolic evidence has largely been generated in postoperative HSCR cohorts or in patients with a history of HAEC. It remains unclear whether preoperative fecal dysbiosis is accompanied by alterations in SCFAs and the broader metabolome before HAEC development. Prospective studies integrating preoperative fecal microbial and metabolic profiling with longitudinal follow-up are needed to investigate whether a coordinated microbial–metabolic phenotype is detectable before surgery and associated with subsequent postoperative HAEC. Therefore, we hypothesized that children with HSCR who later develop postoperative HAEC harbor distinct preoperative microbial and metabolic signatures. To test this hypothesis, we performed a prospective cohort study in children with short-segment HSCR (S-HSCR), integrating 16S rRNA gene sequencing, targeted measurement of fecal short-chain fatty acids (SCFAs), and untargeted fecal metabolomic profiling with 3 years of postoperative follow-up for HAEC occurrence.

## 2 Materials and methods

### 2.1 Study design and patient cohort

Children diagnosed with S-HSCR who underwent surgical treatment at our center between July 2017 and December 2020 were enrolled in this study. Medical history and clinical data were collected. Patients were excluded if any of the following criteria were met: (1) exposure to antibiotics or probiotics within 2 months before sampling; (2) regular rectal irrigation within 1 month before sampling; (3) long-segment HSCR or total colonic aganglionosis; (4) history of HAEC or gastrointestinal surgery; (5) chromosomal abnormalities or syndromic HSCR; (6) persistent postoperative obstructive symptoms. A schematic flowchart of the patient selection process is presented in Supplementary Figure 1.

Patients were followed up for 3 years after surgery through clinic visits or telephone interviews. Postoperative HAEC diagnosis was based on a standardized clinical scoring system, with a score ≥4 indicating HAEC (Frykman et al., 2018; Kyrklund et al., 2020). Patients were categorized into the HAEC and non-HAEC cohorts based on follow-up results.

This prospective cohort study was approved by the Ethics Committee of Xinhua Hospital, Shanghai Jiao Tong University School of Medicine (XHEC-NSFC-2019-074). Written informed consent was obtained from the parents of participants before enrollment.

### 2.2 Fecal sample collection and microbiome profiling

Fresh preoperative fecal samples (2–3 g) were collected using sterile sampling spoons into sterile containers, with care taken to avoid contact with urine, diaper material, or other nonsterile surfaces. For children unable to defecate spontaneously, freshly passed stool following a glycerin enema was collected. The containers were sealed immediately after collection. Samples were stored at 4 °C within 10 min after defecation, transported under cold-chain conditions, and transferred to a −80 °C freezer within 2 h of collection. Repeated freeze–thaw cycles were avoided during subsequent handling. Total microbial genomic DNA was extracted from 200 mg of each fecal sample using the QIAamp DNA Mini Kit (QIAGEN Sciences, USA) according to the manufacturer's instructions. DNA quality was assessed by 1% agarose gel electrophoresis. Extraction blanks and no-template PCR controls were processed alongside the study samples. Neither control yielded detectable amplification. PCR amplification, library preparation, and sequencing were performed by Shanghai Majorbio Bio-Pharm Technology Co., Ltd. The V3–V4 hypervariable regions of the bacterial 16S rRNA gene were amplified using the universal primers 341F (5′-CCTAYGGGRBGCASCAG-3′) and 806R (5′-GGACTACNNGGGTATCTAAT-3′). Following purification and quantification using the QuantiFluor™-ST blue fluorescence quantification system (Promega, USA), amplicons were pooled at equimolar concentrations. Paired-end sequencing (2 × 300 bp) was performed on the Illumina MiSeq PE300 platform (Illumina, USA).

Raw paired-end sequencing reads were quality-filtered using fastp version 0.20.0(Chen et al., 2018). Reads were truncated at any site receiving an average quality score of < 20 over a 50-bp sliding window. Truncated reads shorter than 50 bp and reads containing ambiguous bases were discarded. Paired-end reads were merged using FLASH version 1.2.11 (Magoč and Salzberg, 2011). Only paired-end reads with an overlap longer than 10 bp and a maximum mismatch ratio of 0.2 were merged; reads that could not be assembled were excluded. Samples were distinguished according to exact barcode matching, with no more than two nucleotide mismatches allowed in primer matching. High-quality, non-redundant sequences were clustered into operational taxonomic units (OTUs) at a 97% similarity threshold using UPARSE version 7.0.1090, with chimeric sequences removed during clustering (Edgar, 2013). QIIME version 1.9.1 was used for sequence filtering and OTU-table construction. Taxonomic classification of OTUs was performed using the RDP Classifier algorithm against the SILVA 138 database (http://www.arb-silva.de), with a confidence threshold of 0.7 (Wang et al., 2007). Rarefaction curves were generated to confirm adequate sequencing depth. Bioinformatic analyses were performed using the Majorbio Cloud Platform (https://cloud.majorbio.com).

### 2.3 Targeted quantification of fecal SCFA concentrations

Eight SCFAs, including acetic acid, propionic acid, isobutyric acid, butyric acid, isovaleric acid, valeric acid, hexanoic acid, and isohexanoic acid, were quantified using gas chromatography-mass spectrometry (GC–MS). In addition, fecal samples from 62 age-matched healthy controls were analyzed using the same protocol to further compare SCFA profiles among non-HAEC patients, HAEC patients, and healthy controls. Briefly, 20 mg of each fecal sample was homogenized in 0.5% phosphoric acid and extracted with 0.2 mL n-butanol containing 2-ethylbutyric acid (10 μg/mL) as an internal standard. Analysis was conducted using an Agilent 8890B gas chromatograph in combination with an Agilent 5977B/7000D mass-selective detector. Data acquisition and processing were performed using MassHunter Quantitative Analysis software version 10.0.707.0 (Agilent Technologies, USA).

### 2.4 Untargeted fecal metabolome profiling

Untargeted metabolomic profiling was conducted utilizing an AB Sciex TripleTOF 5600™ LC–MS system (AB SCIEX, USA). Fecal aliquots (50 mg) were extracted with a methanol: water mixture (4:1, v/v) containing L-2-chlorophenylalanine as an internal standard. After ultrasonication, cold incubation at −20 °C, and centrifugation, the supernatants were subjected to LC–MS analysis in both positive and negative electrospray ionization modes. Pooled quality-control samples were prepared by combining equal-volume aliquots of all sample extracts. Each pooled QC sample was processed and analyzed using the same procedure and injection volume as the study samples. One pooled QC sample was injected after every five study samples throughout the analytical sequence to monitor signal stability and analytical reproducibility.

Raw spectra were processed using Progenesis QI software (Waters Corporation, USA) for peak detection, alignment, and annotation. The internal standard and any known artificial peaks were removed. Annotation of the metabolites was performed using the HMDB (http://www.hmdb.ca) and METLIN (https://metlin.scripps.edu) databases. Metabolic features detected in more than 80% of the samples were retained. The internal standard was used to monitor analytical reproducibility, and variables with a relative standard deviation greater than 30% across pooled QC samples were excluded before statistical analysis.

### 2.5 Bioinformatic and statistical analysis

Statistical analyses were performed using GraphPad Prism version 10.4.0 and R software version 4.4.0. The Shapiro–Wilk test was used to evaluate the normality of continuous variables. Normally distributed continuous variables were presented as mean ±SD and compared using Student's t-test. Non-normally distributed continuous variables were presented as medians with interquartile ranges (IQRs) and compared using the Wilcoxon rank-sum test. Categorical variables were compared using the chi-square test. For analyses involving multiple comparisons, *P*-values were adjusted using the Benjamini–Hochberg method where indicated and are reported as Q-values. Because the modest sample size limited statistical power, *Q* < 0.10 was adopted as a threshold to balance false-discovery-rate control against the risk of overlooking potentially informative biological signals for the genus-level differential-abundance analyses performed using MaAsLin2 and the Wilcoxon rank-sum test. All other multiple-comparison analyses were evaluated using *Q* < 0.05 as statistical criteria (Durack et al., 2018; Dwiyanto et al., 2023; Hoskinson et al., 2023).

Alpha diversity, including the Shannon, Simpson, Chao1, and PD whole-tree indices, was calculated at the OTU level using Mothur version 1.48.0. The Gut Microbiome Health Index (GMHI) was calculated to assess the overall state of microbiome health. The core concept of GMHI is to assess the balance between two sets of microbial features that are relatively enriched and depleted in a reference group, respectively (Gupta et al., 2020; Zhu et al., 2025). In the present study, this framework was applied to OTU-level abundance profiles derived from 16S rRNA gene sequencing. The Microbiome Dysbiosis Index (MDI) was calculated to characterize dysbiosis (Xu et al., 2020). Beta diversity was visualized using principal coordinate analysis (PCoA) based on Bray–Curtis distances, and differences in community composition were tested using permutational multivariate analysis of variance (PERMANOVA). Relative abundances of the dominant phyla and top 20 genera were visualized using bar plots. Linear discriminant analysis effect size (LEfSe) was used to identify discriminative taxa, with LDA scores >2.5 and P < 0.05 considered significant (Segata et al., 2011).

To account for potential confounders, PERMANOVA was used to assess the influence of clinical metadata on microbial community structure. For genus-level differential-abundance analysis, MaAsLin2 (Multivariable Association with Linear Models) was fitted with postoperative HAEC status as the variable of interest, while age at surgery (months; continuous), feeding pattern (breastfeeding, formula, or mixed; categorical), and delivery mode (vaginal or cesarean; binary) were entered simultaneously as covariates (Mallick et al., 2021). Cohort status was coded such that positive coefficients indicated higher genus abundance in the non-HAEC cohort relative to the HAEC cohort. To evaluate the predictive potential of preoperative microbial signatures, an exploratory random forest classification model was constructed using the *randomForest* R package with 10-fold cross-validation. Feature importance in the genus-only model was ranked by mean decrease in accuracy, and model performance was assessed by the area under the receiver operating characteristic curve (AUC), sensitivity, and specificity. An additional random forest model incorporating feeding pattern alongside the nine selected microbial genera was constructed to explore whether adding feeding information improved discrimination of postoperative HAEC. Microbial co-occurrence networks were generated using Spearman's rank correlations and visualized in Cytoscape version 3.10.1. Only robust and statistically significant correlations were retained (|r| > 0.5 and Q < 0.05). Network topology was characterized using betweenness centrality, closeness centrality, and degree. Node-level distributions of these metrics were compared between the two networks using Wilcoxon rank-sum tests.

Raw fecal SCFA concentrations were log2-transformed before assessing their associations with the relative abundances of key bacterial genera using Spearman's rank correlation. *P*-values from all pairwise genus–SCFA correlation tests were adjusted together using the Benjamini–Hochberg method. For untargeted metabolomic data, multivariate modeling was initially attempted using the *ropls* R package version 1.6.2. However, because the OPLS-DA model did not pass permutation validation, the model and its VIP values were not used for metabolite selection. Benjamini–Hochberg correction was applied across all detected metabolites, and adjusted *P*-values are reported as Q-values. Candidate metabolites were prioritized using an exploratory univariate screen requiring an FC ≥ 1.2 or ≤ 0.83 and a nominal P < 0.05 from the Wilcoxon rank-sum test (Diaz-Ruiz et al., 2021; Pathmasiri et al., 2024; Duan et al., 2025). Exploratory pathway enrichment and topology analyses of the nominal candidate metabolites were performed using MetaboAnalyst 5.0 (Pang et al., 2022). The pathways are shown by *p*-values from the pathway enrichment analysis (y-axis) and pathway impact values from the pathway topology analysis (x-axis), with the most affected pathway highlighted in red.

## 3 Results

### 3.1 Demographic and clinical characteristics

A total of 62 children with S-HSCR were enrolled in this prospective cohort. During the 3 year postoperative follow-up, 27 patients experienced at least one episode of HAEC (HAEC cohort), whereas 35 remained free of HAEC (non-HAEC cohort). Baseline clinical characteristics were comparable between the two groups, except for a higher proportion of breastfeeding in the non-HAEC cohort compared with the HAEC cohort (*P* = 0.027; Supplementary Table 1).

### 3.2 Preoperative fecal microbial diversity and microbiome health indices were reduced in the HAEC cohort

Sequencing of the 62 fecal samples generated 3,001,122 high-quality reads, with an average read length of 421.36 bp. After quality control and clustering at 97% similarity, 2,197 OTUs were identified, including 1,803 in the non-HAEC cohort, 1,186 in the HAEC cohort, and 792 shared by both cohorts.

Alpha diversity analysis revealed significant differences between the two cohorts (Figure 1A). The HAEC cohort exhibited a significantly reduced Shannon index (*P* = 0.042) and PD whole-tree index (*P* = 0.040), and a significantly elevated Simpson index (*P* = 0.020). These suggested reduced community diversity among patients predisposed to HAEC. The Chao1 index, which reflects the community richness, did not differ significantly between the two cohorts.

**Figure 1:**
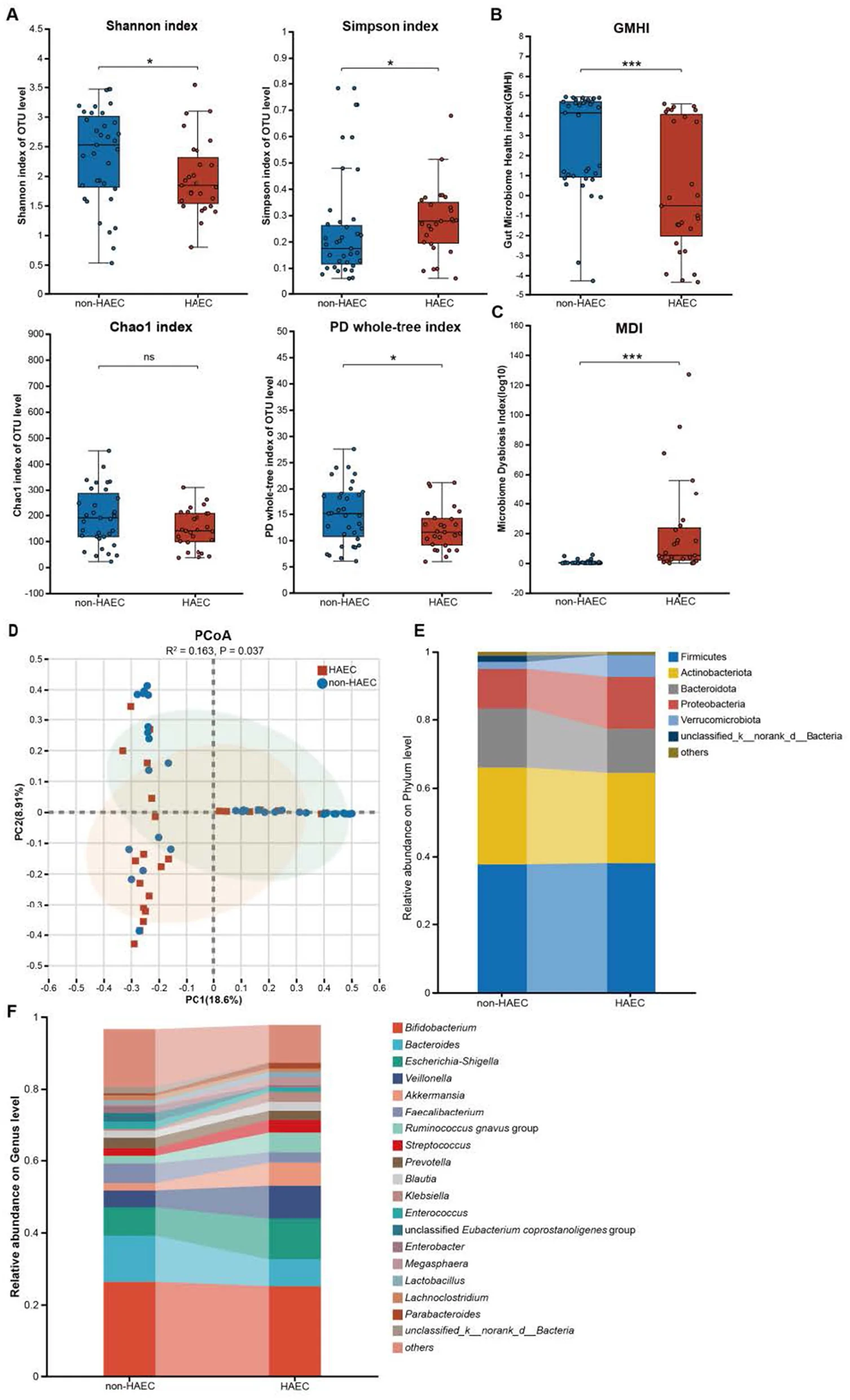
Preoperative gut microbial diversity and global community structure in the non-HAEC and HAEC cohorts. **(A)** Alpha diversity, measured by the Shannon, Simpson, Chao1, and PD whole-tree indices, in the non-HAEC (*n* = 35) and HAEC (*n* = 27) cohorts. **(B, C)** Comparisons of the OTU-based Gut Microbiome Health Index (GMHI; B) and Microbiome Dysbiosis Index (MDI; C) between the two cohorts. **(D)** Principal coordinate analysis (PCoA) plot based on Bray–Curtis distances showing a significant difference in microbial community composition between the two cohorts (PERMANOVA: R^2^ = 0.163, *P* = 0.037). **(E, F)** Relative abundances of the dominant bacterial phyla **(E)** and the top 20 bacterial genera **(F)**. *P < 0.05, ***P < 0.001, assessed by the Wilcoxon rank-sum test.

The OTU-based GMHI was higher in the non-HAEC cohort than in the HAEC cohort (P < 0.001; Figure 1B). To determine the degree of microbial dysbiosis, the MDI was calculated (Figure 1C). The MDI was significantly elevated in the HAEC group (P < 0.001), indicating baseline microbial dysbiosis in patients who subsequently developed HAEC. PCoA based on Bray–Curtis distances further demonstrated a significant difference in global microbial community structure between the two cohorts (*R*^2^ = 0.163, *P* = 0.037; Figure 1D).

### 3.3 Taxonomic composition identified distinct microbial signatures associated with postoperative HAEC

At the phylum level, Firmicutes dominated the microbiota in both cohorts, followed by Actinobacteriota and Bacteroidota (Figure 1E). At the genus level, *Bifidobacterium* was the most abundant taxon overall. Compared with the non-HAEC cohort, the HAEC cohort showed higher relative abundances of *Escherichia-Shigella, Veillonella*, and *Akkermansia*, whereas *Bacteroides* and *Faecalibacterium* were relatively enriched in the non-HAEC cohort (Figure 1F).

To identify discriminative taxonomic biomarkers, LEfSe analysis was performed (Supplementary Figure 2 and Supplementary Table 2). Differences at the phylum level were not significant. At the genus level, 50 bacterial taxa showed different relative abundances between the two cohorts (LDA score > 2.5, *P* < 0.05). The HAEC-associated microbiome was characterized by enrichment of genera such as *Escherichia-Shigella, Oscillibacter, Megasphaera*, and *Rothia*, along with marked depletion of several Firmicutes-associated genera with potential SCFA-producing capacity, including *Subdoligranulum, Eubacterium hallii* group*, Lachnospira, Ruminococcus*, and *Roseburia*. Wilcoxon rank-sum testing further supported the enrichment of six genera in the non-HAEC cohort: *Subdoligranulum, Eubacterium hallii* group, *Fusicatenibacter*, Butyricicoccus, *Monoglobus*, and *Erysipelotrichaceae UCG-003*, which aligned with the LEfSe results (Figure 2A and Supplementary Table 3).

**Figure 2:**
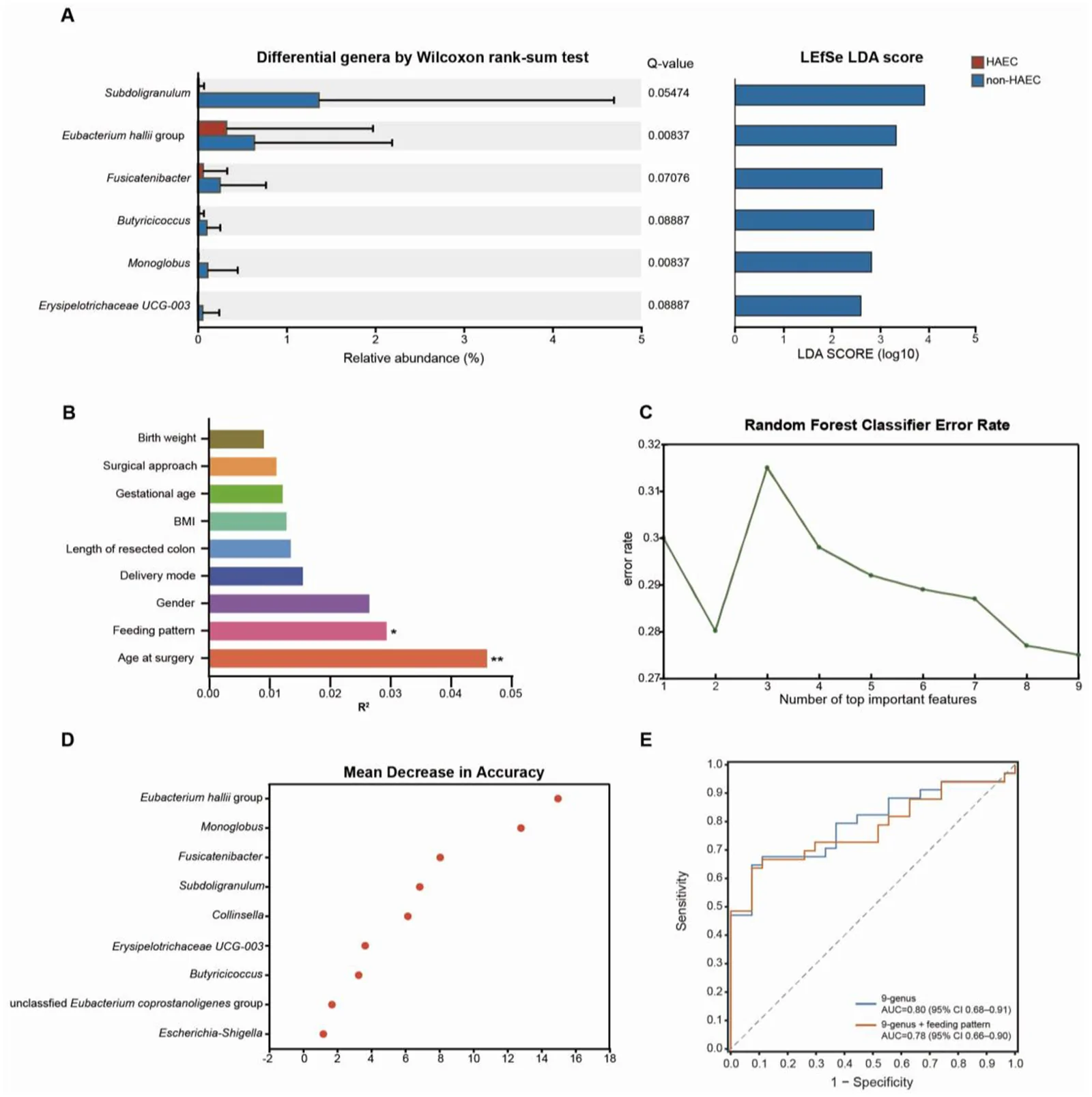
Differential microbial signatures and their predictive potential for postoperative HAEC. **(A)** Genus-level taxa significantly enriched in the non-HAEC cohort (*Q* < 0.10), with corresponding LEfSe linear discriminant analysis (LDA) scores (LDA>2.5). **(B)** PERMANOVA analysis evaluating the contribution of clinical variables to overall microbial community composition, identifying age at surgery and feeding pattern as significant covariates. **(C)** Error rate of the genus-only random forest classifier according to the number of top-ranked microbial features included. **(D)** Top nine candidate microbial biomarkers ranked by feature importance, measured as mean decrease in accuracy in the genus-only model. **(E)** Receiver operating characteristic (ROC) curves for the nine-genus random forest model (AUC = 0.80, 95% *CI*: 0.68–0.91) and the model incorporating the same nine genera plus feeding pattern (AUC = 0.78, 95% CI: 0.66–0.90). *P < 0.05, **P < 0.01.

PERMANOVA indicated that age at surgery and feeding pattern significantly influenced microbial composition (Figure 2B). After adjustment for age at surgery, feeding pattern, and delivery mode in the MaAsLin2 analysis, six genera remained enriched in the non-HAEC cohort at the exploratory Q < 0.10 threshold: *Monoglobus, Fusicatenibacter, Subdoligranulum, Eubacterium hallii* group, an unclassified *Eubacterium coprostanoligenes* group, and *Collinsella* (Supplementary Table 4).

### 3.4 Preoperative microbial signatures showed predictive potential for postoperative HAEC

To evaluate the predictive capacity of preoperative microbial signatures, a random forest classification model was constructed using differentially abundant genera identified by MaAsLin2 and LEfSe. Escherichia-Shigella was enriched in the HAEC cohort in the LEfSe analysis but did not meet the significance threshold in the Wilcoxon analysis and was not among the significant genera identified by covariate-adjusted MaAsLin2. It was nevertheless retained as a *post hoc*, literature-informed exploratory candidate, based on previous clinical and experimental evidence implicating Escherichia in HAEC-associated dysbiosis and intestinal barrier dysfunction (Li et al., 2023). Model performance was optimized when the top nine features were included, as indicated by the lowest classification error (Figure 2C). Feature importance analysis based on mean decrease in accuracy in the genus-only model identified *Eubacterium hallii* group, *Monoglobus*, and *Fusicatenibacter* as the top contributors to model performance (Figure 2D). The nine-genus model achieved an internally cross-validated AUC of 0.80 (95% CI: 0.68–0.91), with a sensitivity of 65% and specificity of 93%. Incorporating feeding pattern alongside these nine genera yielded an AUC of 0.78 (95% CI: 0.66–0.90) (Figure 2E). To assess sensitivity to the *post hoc* inclusion of *Escherichia-Shigella*, an additional eight-genus random forest model was fitted after excluding this genus from the nine-genus feature set. The eight-genus model yielded an AUC of 0.78 (95% CI: 0.63–0.92). When feeding pattern was included, the AUC was 0.77 (95% CI: 0.60–0.88) (Supplementary Figure 3).

### 3.5 The HAEC-associated microbiome exhibited altered co-occurrence network architecture

Genus-level co-occurrence networks were constructed to characterize bacterial interaction patterns in the non-HAEC and HAEC cohorts (|r|>0.5, *Q* < 0.05). The network in non-HAEC was highly interconnected, with 43 nodes and 234 edges. It featured extensive intra-phylum correlations within Firmicutes as well as frequent inter-phylum associations involving Bacteroidota and Proteobacteria. In contrast, the network in HAEC was dramatically different, consisting of 39 nodes and 129 edges, and showed fewer inter-phylum associations (Figure 3A).

**Figure 3:**
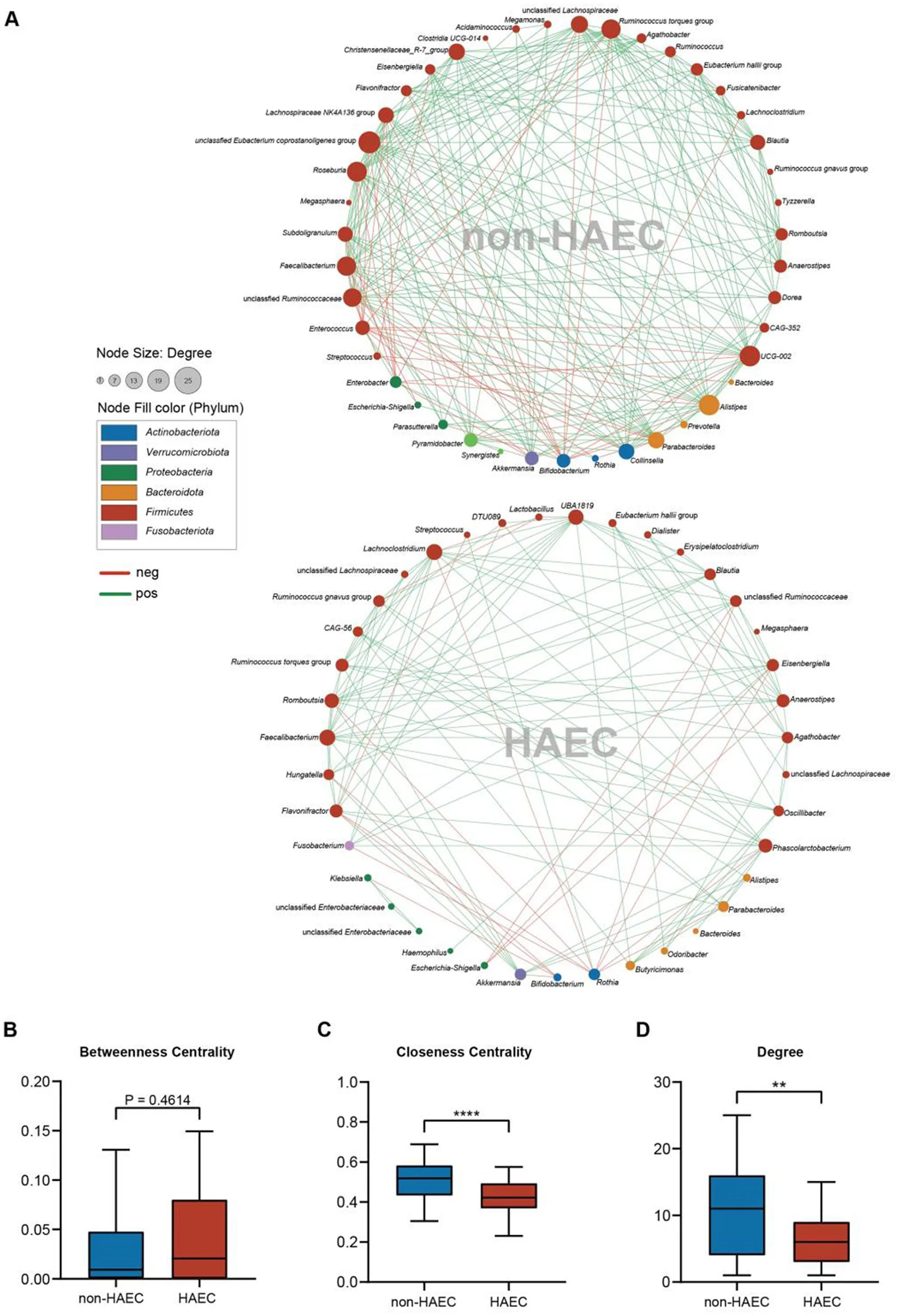
Altered microbial co-occurrence network architecture associated with postoperative HAEC. **(A)** Genus-level co-occurrence networks for the non-HAEC and HAEC cohorts, constructed using significant Spearman correlations (|r| > 0.5, *Q* < 0.05). Nodes represent bacterial genera, with node size proportional to degree centrality and color denoting phylum-level taxonomy. Red and green edges indicate negative and positive correlations, respectively. **(B–D)** Comparison of network topological parameters, including betweenness centrality, closeness centrality, and degree, between the two cohorts. **P < 0.01, ****P < 0.0001, assessed by the Wilcoxon rank-sum test. These tests do not account for node non-independence.

We further quantified network topology using betweenness centrality, closeness centrality, and degree. The two cohorts showed similar levels of betweenness centrality (*P* = 0.461). However, the HAEC cohort exhibited significantly lower closeness centrality (*P* < 0.0001) and degree (P < *0.01*), indicating a less cohesive and potentially less resilient microbial network architecture (Figures 3B–D).

### 3.6 HAEC was associated with reduced fecal SCFAs before surgery

Given that several Firmicutes-associated taxa depleted in the HAEC cohort have potential SCFA-producing capacity, targeted quantification of fecal SCFAs was performed to assess functional metabolic alterations. Compared with the non-HAEC cohort, the HAEC cohort exhibited significantly lower preoperative concentrations of butyric acid, valeric acid, and total SCFAs (Figures 4A–I). No significant between-cohort differences were observed for acetic acid, propionic acid, isobutyric acid, isovaleric acid, hexanoic acid, or isohexanoic acid. Compared with age-matched healthy controls (*n* = 62), the HAEC cohort exhibited significantly lower levels of acetic acid, propionic acid, butyric acid, isobutyric acid, valeric acid, isovaleric acid, and total SCFAs. The non-HAEC cohort also showed significant reductions in acetic acid, propionic acid, butyric acid, isobutyric acid, isovaleric acid, and total SCFAs, indicating a disease-wide baseline depletion of fecal SCFAs in children with S-HSCR (Supplementary Figure 4).

**Figure 4:**
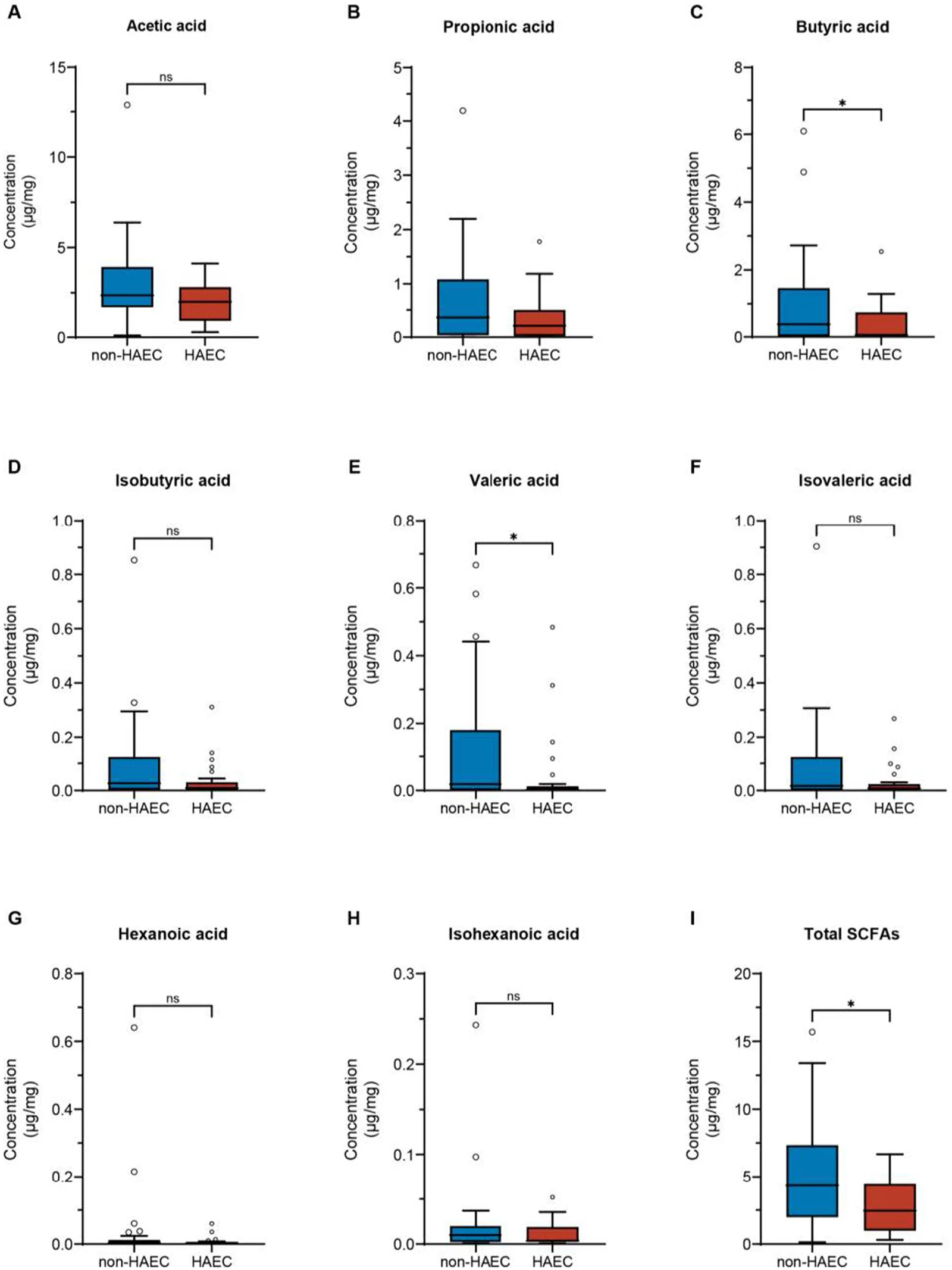
Preoperative fecal short-chain fatty acid profiles in the non-HAEC and HAEC cohorts. **(A–I)** Tukey box-and-whisker plots showing fecal concentrations of acetic acid, propionic acid, butyric acid, isobutyric acid, valeric acid, isovaleric acid, hexanoic acid, isohexanoic acid, and total SCFAs in the non-HAEC and HAEC cohorts. *P < 0.05, assessed by the Wilcoxon rank-sum test.

To further examine the relationship between gut microbial alterations and fecal SCFA profiles, Spearman's rank correlation analysis was performed to assess associations between the nine selected bacterial genera and nine fecal SCFA measures (Figure 5). Several genera enriched in the non-HAEC cohort, including *Eubacterium hallii* group, *Fusicatenibacter, Monoglobus, Butyricicoccus, Subdoligranulum, Collinsella*, and an unclassified *Eubacterium coprostanoligenes* group, showed significant positive correlations with fecal butyric acid levels (all *Q* < 0.001). Among these genera, *Butyricicoccus* was the only taxon positively correlated with total SCFA levels (*Q* < 0.001). In contrast, *Escherichia-Shigella* exhibited negative correlations with isovaleric acid, hexanoic acid, and isohexanoic acid (all *Q* < 0.001).

**Figure 5:**
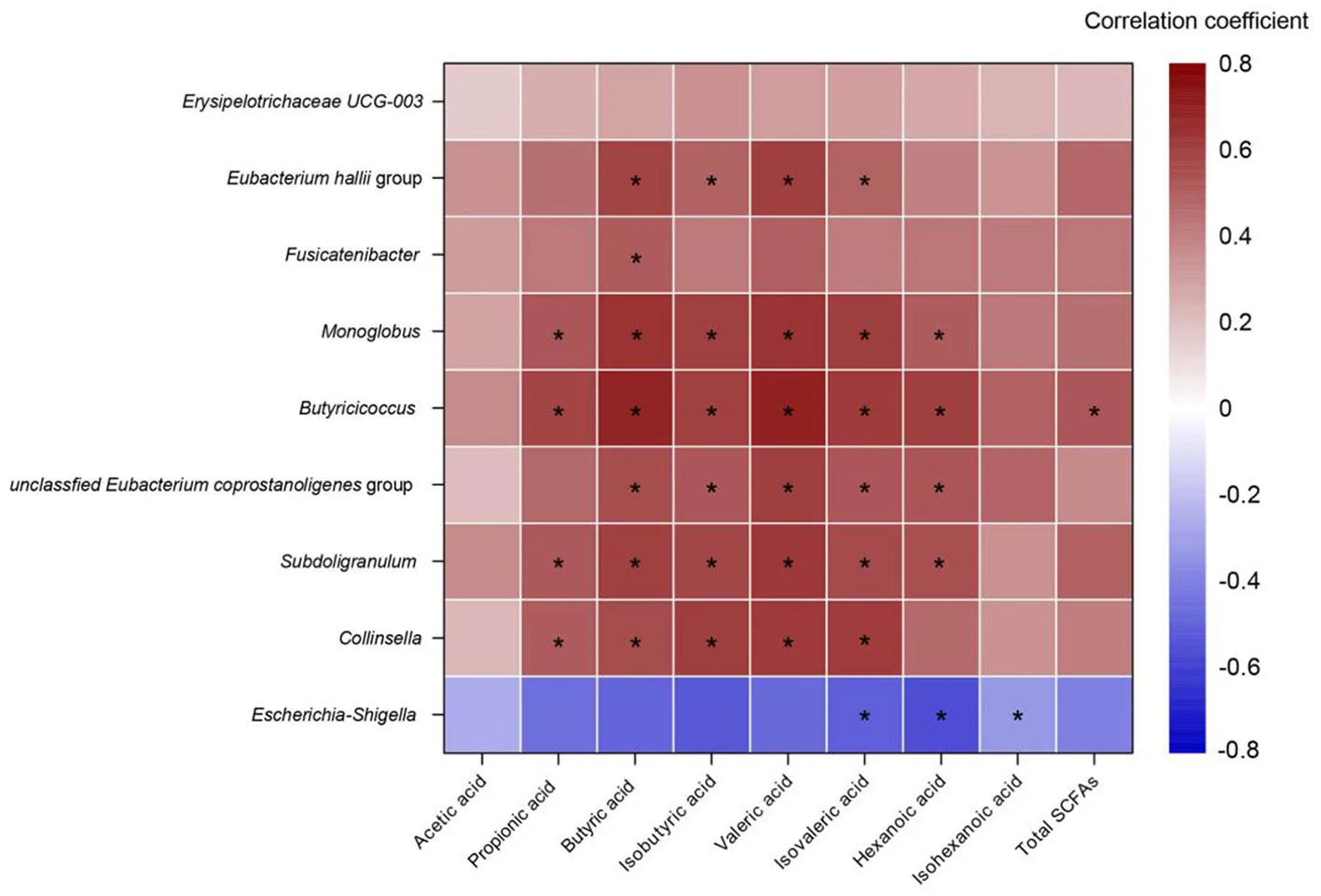
Associations between key differential genera and fecal SCFA profiles. Heatmap showing all 81 pairwise Spearman correlations between the relative abundances of nine selected bacterial genera and nine fecal SCFA measures, including eight individual SCFAs and total SCFAs. Red and blue indicate positive and negative correlations, respectively, with color intensity reflecting the magnitude of Spearman's correlation coefficient. SCFA concentrations were log2-transformed before analysis. P-values were adjusted across all 81 comparisons using the Benjamini–Hochberg method. Asterisks (*) mark correlations meeting both |r| > 0.5 and *Q* < 0.05.

### 3.7 Exploratory untargeted metabolomic profiling identified candidate signals between the non-HAEC and HAEC cohorts

To explore broader functional metabolic shifts beyond SCFAs, untargeted fecal metabolomic profiling was conducted. A total of 1110 metabolites were detected, including 538 and 572 metabolites in positive and negative ion modes, respectively. The principal component analysis (PCA) score plot (Supplementary Figure 5A) showed substantial overlap between the two cohorts, and PERMANOVA indicated a modest difference in overall metabolomic profiles between the non-HAEC and HAEC cohorts (*R*^2^ = 0.045, *P* = 0.022).

Univariate analysis identified 86 nominal candidate metabolites meeting the stated fold-change and nominal *P*-value criteria, including 78 metabolites with higher and 8 with lower abundance in the non-HAEC cohort (Supplementary Figure 5B and Supplementary Table 5). After Benjamini–Hochberg correction, none of these 86 candidates remained statistically significant at *Q* < 0.05 (*Q*-value range, 0.255–0.307). Exploratory pathway enrichment analysis of the nominal candidates yielded annotations involving penicillin and cephalosporin biosynthesis, caffeine metabolism, retinol metabolism, lysine biosynthesis, biosynthesis of alkaloids derived from histidine and purine, cortisol synthesis and secretion, ovarian steroidogenesis, and Cushing syndrome (Supplementary Figure 5C and Supplementary Table 6). Five metabolic pathways displayed lower *P*-values and higher impact in the pathway analysis between the non-HAEC and HAEC cohorts, including pathways related to lysine biosynthesis, lysine degradation, pyrimidine metabolism, steroid hormone biosynthesis, and caffeine metabolism (Supplementary Figure 5D). Because no individual metabolite survived Benjamini–Hochberg correction, these pathway outputs were considered exploratory and hypothesis-generating rather than statistically confirmed pathway alterations.

## 4 Discussion

Postoperative HAEC remains a major concern after the pull-through procedure. Despite the established risk factors for postoperative obstructive complications, including trisomy 21 and long-segment aganglionosis, a considerable number of patients develop HAEC without these conditions. There is growing support for a multifactorial model in which ENS dysfunction, microbial dysbiosis, intestinal barrier failure, and irregular mucosal immune responses interact to promote HAEC development (Li et al., 2023; Ziogas et al., 2024). Studies in HSCR animal models have shown that ENS dysfunction can disrupt intestinal motility, barrier function, and host-microbe interactions, thereby triggering early intestinal inflammation and microbial dysbiosis (Cheng et al., 2018; Touré et al., 2019; Arnaud et al., 2021). Clinical research has revealed that the gut microbiome in individuals with HSCR differs from that of healthy individuals, and that those with HAEC may exhibit additional microbial alterations (Frykman et al., 2015; Li et al., 2016; Arnaud et al., 2022; Biassoni et al., 2022; Zhan et al., 2022). However, a major limitation of many prior studies is the timing of sample collection. The biological onset of HAEC may precede its clinical manifestation. By the time an episode is clinically diagnosed and a fecal sample can be obtained, active inflammation, altered intestinal transit or stasis, changes in feeding, rectal irrigation, antibiotic exposure, or other therapeutic interventions may already have reshaped the intestinal microbial and metabolic environment. Consequently, samples collected during or after a clinically recognized episode may not faithfully represent the state immediately preceding HAEC onset, making it difficult to distinguish predisposing alterations from consequences of active disease or its treatment.

The early-life gut microbiome plays a critical role in immune maturation and long-term intestinal homeostasis, and perturbations during this developmental window may exert long-lasting effects on later disease susceptibility (Gensollen et al., 2016; Stinson et al., 2017; Guo et al., 2021; Henrick et al., 2021). Accumulating evidence indicates that the gut microbiome and its metabolites can modulate ENS development, neuronal maturation, plasticity, and regeneration through immune-mediated pathways, host receptor activation, and crosstalk with intestinal epithelial and enteroendocrine cells, particularly during the postnatal period when microbial colonization and ENS development occur in parallel (Joly et al., 2021; Vicentini et al., 2021). Conversely, a well-functioning ENS promotes intestinal health by modulating the dynamic balance between pro- and anti-inflammatory microbial communities (Rolig et al., 2017). In HSCR, ENS abnormalities are present from birth and may influence intestinal motility, luminal stasis, host–microbe interactions, and microbial maturation from the earliest postnatal stages (Ward et al., 2012; Ziogas et al., 2024). Congenital ENS defects may also disrupt mucosal immune homeostasis and shape the gut microbiota through impaired neuroimmune signaling and altered cholinergic regulation (Touré et al., 2019). These alterations have been linked to imbalanced macrophage polarization and a pro-inflammatory mucosal environment, which may disrupt early microbial colonization, facilitate intestinal dysbiosis, and increase HAEC susceptibility(Müller et al., 2022; Ji et al., 2023; Zheng et al., 2025b). Consistent with this concept, HAEC-associated microbial dysbiosis appears most pronounced in children younger than 2 years (Arnaud et al., 2022). Likewise, postoperative HSCR cohorts exhibit long-term alterations in the developmental trajectories of the gut microbiome rather than simple normalization after surgery (Murphy et al., 2025). In children with a history of HAEC, baseline microbial dysbiosis is more severe (Neuvonen et al., 2018). In addition, the histological grade of preoperative intestinal inflammation has been shown to be closely associated with an increased risk of postoperative HAEC (Cheng et al., 2017). Together, these findings suggest that microbial alterations relevant to HAEC may arise early in life and continue to shape the intestinal ecosystem thereafter.

In the present study, children who subsequently developed postoperative HAEC had a less diverse and more dysbiotic gut ecosystem before surgery, characterized by lower alpha diversity, reduced GMHI, and increased MDI. Greater microbial diversity is generally considered important for maintaining intestinal homeostasis, whereas loss of ecological balance may contribute to HAEC development (Frykman et al., 2015). Previous animal and clinical studies have confirmed that HSCR is associated with gut dysbiosis, with significantly reduced alpha diversity compared with healthy controls (Cheng et al., 2018; Neuvonen et al., 2018; Chantakhow et al., 2021; Tian et al., 2023; Shi et al., 2024). Longitudinal microbiome analysis has shown that alpha diversity in patients with HSCR increases significantly by 6 months postoperatively compared with the time of initial diagnosis, suggesting a partial shift toward microbial re-equilibration after definitive surgery (Chantakhow et al., 2026). A prospective study also reported reduced alpha diversity in patients who subsequently developed postoperative HAEC when evaluating the relationship between the enteric microbiome at the time of surgery and later HAEC risk (Tang et al., 2020). A retrospective study showed that alpha diversity was lower in children with HAEC than in children with HSCR without HAEC (Xie et al., 2022). Children with total colonic aganglionosis, a condition associated with the highest risk of HAEC, also exhibit significantly reduced gut microbial alpha diversity compared with those with S-HSCR (Pini Prato et al., 2019). Overall, these data support the hypothesis that reduced gut microbial diversity and a pre-existing dysbiotic state are closely associated with increased susceptibility to HAEC.

Taxonomically, our analysis revealed that the non-HAEC group was predominantly enriched in beneficial SCFA-associated genera from the Lachnospiraceae and Ruminococcaceae families, including the *Eubacterium hallii* group, *Butyricicoccus, Subdoligranulum, Fusicatenibacter*, and *Monoglobus*. Previous cross-sectional studies have reported that, compared with patients with HSCR alone, those with a history of HAEC showed reduced Firmicutes abundance (Frykman et al., 2015; Li et al., 2016; Pini Prato et al., 2019). Most studies in HSCR animal models also observed a significant decrease in Firmicutes (Chantakhow et al., 2021). As key contributors to saccharolytic fermentation and SCFA production, these anaerobic commensals play important roles in fortifying the intestinal epithelial barrier and exerting anti-inflammatory effects (Schirmer et al., 2019; Siddiqui and Cresci, 2021). Feature importance analysis identified *the Eubacterium hallii group and Monoglobus as the top-contributing* taxa in the random forest model. The *Eubacterium hallii group* is a recognized SCFA-producing commensal associated with butyrate and propionate production and anti-inflammatory benefits (Mukherjee et al., 2020; Lee and Hwang, 2025). Recent longitudinal work in HSCR has shown that recovery of *Eubacterium* and *Eubacteriales* accompanies postoperative microbial re-equilibration (Chantakhow et al., 2026). *Monoglobus* is a specialized pectin-degrading bacterium involved in complex polysaccharide fermentation and maintenance of mucosal homeostasis (Kim et al., 2019). Depletion of these taxa in the HAEC cohort suggests that children predisposed to postoperative HAEC may already harbor a preoperative ecosystem with reduced ecological resilience and diminished capacity to support mucosal homeostasis (Zhang et al., 2022).

In contrast, the HAEC-associated microbiota was characterized by enrichment of *Escherichia–Shigella, Megasphaera*, and *Rothia*. Among these taxa, *Escherichia-Shigella* remains a biologically plausible exploratory candidate; however, its *post hoc* inclusion and the small numerical differences in AUC observed after its exclusion do not establish its incremental predictive value in this cohort. *Escherichia-Shigella* is an opportunistic pathogenic taxon within the Enterobacteriaceae family. Existing clinical studies have implicated expansion of Proteobacteria, especially *Escherichia and* Enterobacteriaceae, in HAEC pathogenesis, potentially through upregulation of lipopolysaccharide biosynthesis and enrichment of microbial virulence-associated traits such as fimbriae and secretion systems (Li et al., 2016; Neuvonen et al., 2018; Tang et al., 2020; Arbizu et al., 2021; Biassoni et al., 2022; Barmadisatrio et al., 2025). In animal models of rectosigmoid hypoganglionosis, ENS disruption drives progressive alterations in the gut microbiome and impairs epithelial barrier function, characterized by increased Proteobacteria abundance and decreased Firmicutes abundance (Arnaud et al., 2021; Budianto et al., 2024). Experimental evidence from HSCR animal models suggests that mucosal barrier dysfunction and microbial dysbiosis may precede the onset of HAEC, as reflected by decreased luminal levels of secretory phospholipase A2 and increased intestinal invasion by Escherichia coli (Pierre et al., 2014). As intestinal stasis progresses, Escherichia coli may translocate to extraintestinal sites more frequently than other bacteria, in association with impaired mucosal barrier function and bowel inflammation (Mitroudi et al., 2019). *Escherichia* may also activate TLR4/p-p38/NF-κB signaling and disrupt intestinal mucosal integrity in HSCR animal models (Zheng et al., 2022). These findings support the possibility that overabundance of *Escherichia-Shigella* in the HAEC cohort reflects a pre-existing pro-inflammatory microbial state that may contribute to HAEC development.

These taxonomic changes also showed potential predictive value. We constructed a random forest classifier that achieved an AUC of 0.80. Similarly, Tang et al. provided prospective evidence that microbial features present before postoperative HAEC could have predictive value, reporting a 21-OTU signature derived from mucosal tissue obtained from the dilated colon at surgery, which could predict postoperative HAEC with an AUC of 0.90 (Tang et al., 2020). More recently, Chantakhow et al. identified region-specific taxa associated with subsequent HAEC in intraoperative proximal and distal intestinal samples (Chantakhow et al., 2026). These studies collectively support the concept that microbial alterations may be detectable before postoperative HAEC. However, their specific taxa and estimated model performance should not be directly compared. The cohorts differed in geographic and dietary backgrounds, disease characteristics, perioperative management, sampling sites and time points, taxonomic resolution, bioinformatic pipelines, feature-selection procedures, and validation strategies. A recent nomogram incorporating factors such as preoperative HAEC, bowel preparation status, and disease extent achieved an AUC of 0.79, further supporting the clinical value of risk prediction for postoperative HAEC (Zheng et al., 2025a). Because our cohort was restricted to S-HSCR and excluded children with prior HAEC, the preoperative fecal microbial features identified here may warrant future evaluation as potential adjuncts to conventional clinical risk assessment in children without several major established risk factors. Nevertheless, the present panel remains an exploratory candidate. Its clinical value can only be established through external validation of the same model in larger cohorts and by determining whether it provides a basis for future preoperative risk stratification and targeted preventive strategies.

Beyond taxonomic shifts, the co-occurrence network analysis suggested lower microbial connectivity in the HAEC cohort, with fewer edges and lower observed closeness centrality and degree. These findings imply that the microbiota associated with later HAEC development is characterized not only by depletion of potentially protective taxa but also by weakening of the broader ecological architecture that normally supports inter-taxa interactions and community stability.

In this study, many taxa depleted in the HAEC cohort were functionally linked to microbial fermentation and potential SCFA production. SCFAs are produced predominantly in the colon and serve as critical signaling molecules and metabolic substrates in maintaining gut homeostasis (Tan et al., 2023). SCFAs have also been implicated in the regulation of ENS development and function, suggesting that impaired microbial fermentation may further interact with the underlying enteric neuropathy of HSCR (Montalva et al., 2023). Because prior work demonstrated that several SCFA-associated taxa were significantly reduced and correlated with SCFA levels in patients with HSCR (Liu et al., 2024), we measured fecal SCFAs to assess whether corresponding metabolic abnormalities accompanied the observed taxonomic alterations. Both the HAEC and non-HAEC cohorts showed significantly lower fecal SCFA levels than healthy controls, indicating that HSCR itself may be associated with disease-wide impairment of microbial metabolic output. Children who subsequently developed postoperative HAEC represented a more severely affected subgroup, with significantly lower preoperative concentrations of butyric acid, valeric acid, and total SCFAs than children who remained HAEC-free. Demehri et al. previously reported an approximately fourfold reduction in total fecal SCFAs among children with a history of HAEC, also supporting impaired microbial fermentation in relation to HAEC (Demehri et al., 2016). However, their analysis of relative SCFA composition showed reduced acetate and an increased relative proportion of butyrate, whereas our measurements of absolute concentrations showed lower preoperative butyrate in children who subsequently developed HAEC. These findings should be interpreted as broadly complementary rather than contradictory. Demehri et al. evaluated a cross-sectional cohort stratified by a prior history of HAEC and reported a marked reduction in total fecal SCFAs, with a higher relative proportion of butyrate despite preservation of its absolute concentration. In contrast, our samples were collected preoperatively, before the subsequent development of postoperative HAEC, and showed lower absolute concentrations of butyrate, valerate, and total SCFAs in children who later developed HAEC. Differences in population background, disease stage, sampling time relative to HAEC events, and the reporting of relative vs. absolute SCFA levels may therefore partly account for the apparent differences between studies.

Among the altered SCFAs, butyrate is likely to be of particular biological importance. Butyrate supports colonocyte energy metabolism, epithelial integrity, and mucosal immune regulation (Siddiqui and Cresci, 2021; Zhang et al., 2021; Ornelas et al., 2023). Experimental work in hypoganglionosis has also linked SCFAs to proliferation and neurogenesis of enteric neural crest-derived cells (Tian et al., 2023). In addition, patients with HSCR have an approximately fivefold increased risk of developing inflammatory bowel disease, which is commonly associated with depletion of SCFA-producing bacteria and reduced fecal SCFA levels, especially butyrate (Löf Granström et al., 2018; Parada Venegas et al., 2019). In our cohort, several genera enriched in the non-HAEC cohort, including the *Eubacterium hallii* group and *Butyricicoccus*, were positively correlated with fecal butyrate and total SCFA levels, whereas *Escherichia-Shigella* were inversely correlated with multiple SCFAs. These findings suggest that SCFA depletion, particularly butyrate deficiency, may represent a biologically functional correlate of the preoperative dysbiotic phenotype in children who later developed postoperative HAEC. However, their potential preventive or therapeutic relevance requires longitudinal and interventional validation.

Currently, evidence for the prevention of HAEC with probiotics remains inconsistent, with randomized trials and meta-analyses showing no consistent preventive benefit (Wang et al., 2015; Nakamura et al., 2018; Soh et al., 2018). Future interventions might be function-led rather than based solely on conventional probiotic labels. Candidate strategies could include strain-defined butyrate-producing organisms to restore endogenous butyrate production, as well as controlled butyrate or other postbiotic formulations. Compared with conventional probiotic supplementation alone, such functionally targeted strategies may provide a more biologically relevant direction for future intervention studies informed by the microbial and metabolic disturbances observed in our cohort.

To extend the analysis beyond SCFAs, we performed exploratory untargeted metabolomics to obtain a broader functional landscape of host–microbe metabolic interactions. Exploratory pathway analysis yielded annotations related to lysine biosynthesis and degradation. A Mendelian randomization study suggested that alterations in beneficial gut microbiota may be linked to changes in serum lysine levels in patients with HSCR (Wang et al., 2024). In postoperative patients with HSCR, Plekhova et al. reported alterations in the metabolism of amino sugar, lysine, sialic acid, hyaluronan, and heparan sulfate compared with healthy controls, whereas tyrosine metabolism differed according to the history of HAEC (Plekhova et al., 2021). In a serum metabolomics study of children with HSCR, Yang et al. identified 21 biomarkers and revealed profound disruptions in amino acid pathways, with tryptophan and lysine metabolism emerging as important metabolic signatures (Yang et al., 2023). Previous studies have demonstrated that lysine can serve as an alternative substrate for microbial butyrate production in the human gut, with certain intestinal bacteria capable of converting lysine into butyrate and acetate (Bui et al., 2015). These previous studies provide a rationale for investigating lysine-related metabolism, but they do not validate the exploratory annotations observed in our cohort. Differences in study populations, analytical platforms, and statistical methods also limit direct comparison. The present lysine-related findings should therefore be regarded as hypotheses for further investigation.

Several steroid hormone-related annotations were also obtained in the exploratory pathway analysis, but their biological relevance to HAEC remains uncertain. The penicillin and cephalosporin biosynthesis and caffeine metabolism annotations likewise were not assigned HAEC-specific biological significance. These database-derived annotations do not establish that the corresponding pathways are altered in this cohort. The untargeted metabolomic results therefore provide hypotheses for further investigation rather than evidence of a metabolic mechanism underlying HAEC.

Taken together, our findings support a biologically plausible pathway in which congenital ENS dysfunction and dysmotility may promote luminal stasis and impaired mucosal defense, potentially favoring the loss of anaerobic SCFA-associated commensals, reduced microbial community connectivity, and expansion of pathobionts such as *Escherichia–Shigella*. Lower fecal concentrations of butyrate and other SCFAs may reflect altered microbial fermentation and could plausibly be associated with reduced support for colonocyte metabolism, mucus production, epithelial barrier integrity, and anti-inflammatory immune regulation, whereas pathobiont-derived products and bacterial translocation may activate TLR4/p38/NF-κB signaling and amplify mucosal inflammation (Pierre et al., 2014; Schulthess et al., 2019; Liang et al., 2022; Zheng et al., 2022). These mutually reinforcing disturbances could reduce intestinal ecological resilience and may lower the threshold for surgery-related or other perioperative perturbations to precipitate postoperative HAEC in susceptible children. The lysine-related pathway signals may reflect altered microbial substrate utilization and fermentative metabolism, but they remain hypothesis-generating because they were identified through exploratory untargeted metabolomic analysis. In the present study, we did not perform interventional animal experiments; therefore, the directionality and causal links among dysbiosis, metabolic alterations, barrier or immune dysfunction, and HAEC remain undetermined. Nevertheless, this mechanistic framework remains biologically plausible rather than causally demonstrated by the present study. Translation of these findings into clinical practice will require a stepwise approach. First, the preoperative microbial and SCFA signatures should be externally validated in larger multicenter cohorts using standardized sampling procedures and HAEC definitions, while longitudinal sampling across the perioperative period could clarify their temporal relationship with subsequent HAEC. Integration of shotgun metagenomics or metatranscriptomics with targeted metabolite measurements may further identify the strain-level organisms and metabolic functions underlying these associations. Second, candidate mechanisms and interventions should be evaluated in relevant *ex vivo* and HSCR animal models to determine whether restoring butyrate-producing microbial functions or supplementing with microbial metabolites improves epithelial barrier integrity and attenuates inflammatory responses. Third, promising strains or postbiotic formulations would require rigorous assessment of strain-level safety, dose, formulation, functional target engagement, and biological effects before prospective clinical evaluation.

This study has several limitations. First, the relatively modest sample size limited statistical power, largely due to strict exclusion criteria intended to minimize major confounders, such as recent antibiotic or probiotic exposure. This challenge is further compounded by the substantial inter-individual variability reported in the gut microbiota of children with HSCR (Pini Prato et al., 2019). Because of the limited sample size, we were unable to construct an independent validation cohort for the random forest model. Feature selection, model development, and internal performance assessment were all conducted within the same single-center cohort; the reported AUC may be optimistic, and the robustness of the model remains uncertain. Given the modest cohort size, using Q < 0.05 identified only a limited number of features, potentially reflecting reduced statistical power and an increased risk of false-negative findings. We therefore used Q < 0.10 as an exploratory discovery threshold for microbial differential abundance analyses performed with MaAsLin2 and the Wilcoxon rank-sum test. Although this approach improved sensitivity for candidate identification, it also permits a higher expected proportion of false discoveries. Independent external validation of the candidate microbial features and the exploratory random forest model in larger multicenter cohorts is needed to improve statistical power, enable clinically relevant stratified analyses, and assess generalizability.

Second, feeding patterns differed between the cohorts and may confound the observed microbiome–HAEC associations. Although MaAsLin2 adjusted for age at surgery, feeding pattern, and delivery mode, and an additional random forest model incorporated feeding pattern, these analyses do not establish an independent protective effect of breastfeeding or eliminate residual confounding. Feeding-pattern subgroup analyses were not performed because the breastfeeding, formula-feeding, and mixed-feeding subgroups were relatively small, limiting the reliability of subgroup-specific estimates.

Third, fecal samples were collected at a single preoperative time point. Although the prospective design confirms that the baseline microbial and metabolic profiles were measured before the recorded postoperative HAEC episodes, it did not capture within-patient changes across the perioperative period, at HAEC onset, or during recovery (Parker et al., 2022). The interval between sample collection and HAEC onset varied among individuals, limiting assessment of time-dependent relationships. Longitudinal investigation of HAEC also presents an inherent sampling challenge because its biological onset may precede clinical recognition. By the time an HAEC episode is diagnosed and a fecal sample can be obtained, active inflammation, altered intestinal transit or stasis, changes in feeding, rectal irrigation, antibiotic exposure, or other treatment may already have modified the intestinal microbial and metabolic environment. Therefore, even samples collected during or after a clinically recognized episode may not faithfully represent the state immediately preceding HAEC onset. The signatures identified in the present study should consequently be interpreted as prospective associations and potential markers of susceptibility rather than evidence of a causal role in HAEC development.

In addition, 16S rRNA sequencing limited taxonomic resolution to the genus level and did not permit direct analysis of strain-level variation or functional gene content. The use of 97% identity-based OTU clustering reflects the legacy bioinformatic workflow adopted during the earlier phase of this cohort study, which may obscure fine-scale microbial variation. Consequently, potentially important strain-specific differences in virulence, metabolic capacity, and host interactions could not be assessed. Although targeted SCFA analysis and exploratory untargeted metabolomics provided complementary functional information, we did not perform a formal integrative analysis linking microbial features to global untargeted metabolomic profiles. Healthy controls were included only in the targeted SCFA analysis, limiting our ability to distinguish HAEC-specific alterations from broader S-HSCR abnormalities. The untargeted metabolomics screen relied on nominal *P*-values, increasing the risk of false-positive findings. Targeted metabolomic validation was not performed in the present cohort, and the candidate metabolites and related pathway annotations require independent confirmation. The Wilcoxon rank-sum tests used to compare node-level network metrics did not account for node non-independence, and the observed network differences require confirmation using permutation tests. Future studies should combine shotgun metagenomics and targeted metabolomics with prospectively scheduled serial sampling before surgery and at predefined postoperative intervals, supplemented by episode-associated sampling before treatment whenever clinically feasible. Such longitudinal studies would allow characterization of within-patient microbial and metabolic trajectories and help distinguish alterations that precede clinical HAEC, persist after recovery, or arise as consequences of HAEC and its treatment. Interventional experiments using germ-free or gnotobiotic animal models will also be required to test the causal roles of the candidate microbial and metabolic alterations.

## 5 Conclusion

In conclusion, this prospective study demonstrates that children with S-HSCR who subsequently develop postoperative HAEC exhibit distinct alterations in the gut microbiome and metabolism before surgical intervention. These alterations are characterized by reduced microbial diversity, diminished network cohesion, depletion of beneficial Firmicutes-associated commensals, reduced fecal SCFA levels, and broader exploratory metabolic disturbances. Our findings suggest that postoperative HAEC may be preceded by early disruption of host–microbe metabolic homeostasis and support the potential value of developing preoperative microbiota-based risk stratification and preventive strategies. Larger longitudinal studies with independent validation are required before these microbial and metabolic signatures can inform clinical prediction or microbiota-targeted preventive interventions.

## Data Availability

The 16S rRNA gene sequencing data generated in this study have been deposited in the NCBI Sequence Read Archive (PRJNA1474974). Other data supporting the findings of this study are available from the corresponding author upon reasonable request, subject to ethical and privacy restrictions.

## Supplementary material

Figure S1 Flowchart of patient enrollment and study design. Schematic overview of the study design, detailing patient enrollment, inclusion and exclusion criteria, preoperative fecal sample collection, and the longitudinal three-year postoperative follow-up for HAEC occurrence. S-HSCR, short-segment Hirschsprung disease; L-HSCR, long-segment Hirschsprung disease; TCA, total colonic aganglionosis.

Figure S2 LEfSe-based identification of discriminative bacterial taxa associated with postoperative HAEC. Linear discriminant analysis effect size (LEfSe) bar plot showing genus-level taxa differentially enriched between the non-HAEC and HAEC cohorts. Red and blue bars indicate taxa enriched in the HAEC and non-HAEC cohorts, respectively. Only taxa with an LDA score > 2.5 and *P* < 0.05 are shown.

Figure S3 Sensitivity analysis of random forest models with and without *Escherichia-Shigella*. ROC curves summarize discrimination of postoperative HAEC for four random forest models. The nine-genus models included *Escherichia-Shigella*, whereas the eight-genus models excluded this genus. Blue curves represent models that contain only microbial genera, and orange curves represent models that additionally incorporate feeding patterns. Solid and dashed curves indicate the nine-genus and eight-genus models, respectively. The grey diagonal line indicates chance discrimination. AUC differences are presented descriptively.

Figure S4 Comparison of fecal SCFA concentrations among healthy controls, non-HAEC patients, and HAEC patients. Tukey box-and-whisker plots showing fecal concentrations of acetic acid, propionic acid, butyric acid, isobutyric acid, valeric acid, isovaleric acid, hexanoic acid, isohexanoic acid, and total SCFAs among non-HAEC patients (*n* = 35), HAEC patients (*n* = 27), and healthy controls (*n* = 62). ns, not significant; ^*^*P* < 0.05, ^**^*P* < 0.01, ^***^*P* < 0.001, ^****^*P* < 0.0001, assessed by Kruskal–Wallis test followed by Dunn's post hoc test.

Figure S5 Exploratory untargeted fecal metabolomic analysis in the non-HAEC and HAEC cohorts. **(A)** Principal component analysis (PCA) score plot showing overall metabolomic differences between the non-HAEC and HAEC cohorts (PERMANOVA: *R*^2^ = 0.045, *P* = 0.022). **(B)** Volcano plot illustrating nominal candidate metabolites, defined as fold change (FC) ≥ 1.2 or ≤ 0.83 and nominal *P* < 0.05. Metabolites upregulated and downregulated in the non-HAEC cohort are shown in red and blue, respectively. **(C)** KEGG pathway enrichment analysis of the nominal candidate metabolites. **(D)** Pathway topology analysis highlighting metabolic pathways with relatively high impact values.
