# Supplementary material for: Preoperative gut microbial and metabolic signatures associated with postoperative Hirschsprung-associated enterocolitis in short-segment Hirschsprung disease
Source: Front Microbiol. 2026 Sep 30;17:1911505. doi: 10.3389/fmicb.2026.1911505 (PMC13661467; doi:10.3389/fmicb.2026.1911505)
Supplement: Supplementary file 1 [file Table_1.docx]

| **Table S1. Baseline demographic and clinical characteristics of the non-HAEC and HAEC cohorts** | | | |
| --- | --- | --- | --- |
|  | **non-HAEC**  **(n=35)** | **HAEC**  **(n=27)** | ***P*-value** |
| **Sex (male)** | 20 (57.1%) | 18 (66.7%) | 0.445 |
| **Age at surgery (months)** | 7.1±3.8 | 5.7±2.2 | 0.318 |
| **BMI at surgery (kg/m²)** | 18.2±1.9 | 17.1±1.6 | 0.817 |
| **Birth weight (kg)** | 3.6±0.6 | 3.5±0.4 | 0.812 |
| **Gestational age at birth (weeks)** | 39.3±1.7 | 39.6±1.5 | 0.401 |
| **Mode of delivery (vaginal)** | 18 (51.4%) | 11 (40.7%) | 0.473 |
| **Feeding pattern** |  |  | **0.027** |
| Breastfeeding | 23 (65.7%) | 9 (33.3%) |  |
| Formula | 6 (17.1%) | 6 (22.2%) |  |
| Mixed | 6 (17.1%) | 12 (44.4%) |  |
| **Preoperative bowel management** |  |  | 0.709 |
| Glycerin enema | 26 (74.2%) | 19 (70.4%) |  |
| Glycerin enema + anal dilation | 8 (22.9%) | 6 (22.2%) |  |
| Anal dilation | 1 (2.9%) | 2 (7.4%) |  |
| **Preoperative defecation frequency (times/week)** | 4.8±1.5 | 4.2±1.9 | 0.426 |
| **Surgical approach** |  |  | 0.901 |
| Transanal pull-through | 20 (57.1%) | 15 (55.6%) |  |
| Laparoscopic | 15 (42.9%) | 12 (44.4%) |  |
| **Mean resected bowel length (cm)** | 24.2±8.6 | 21.2±7.8 | 0.411 |
| **Follow-up duration (months)** | 38.2±2.6 | 37.1±2.1 | 0.653 |
| **Age at follow-up (months)** | 46.1±6.6 | 43.2±5.2 | 0.328 |
